# Potentially inappropriate medication use among older adult patients on follow-up at the chronic care clinic of a specialized teaching hospital in Ethiopia. A cross-sectional study

**DOI:** 10.1186/s12877-021-02463-9

**Published:** 2021-10-07

**Authors:** Behailu Terefe Tesfaye, Mihret Terefe Tessema, Mengist Awoke Yizengaw, Dula Dessalegn Bosho

**Affiliations:** 1 School of Pharmacy, Department of Clinical Pharmacy, Institute of Health, Faculty of Health Sciences, Jimma University, P.O.BOX: 378, Jimma, Ethiopia; 2 School of Pharmacy, Institute of Health, Faculty of Health Sciences, Jimma University Medical Center, P.O.BOX: 378, Jimma, Ethiopia

**Keywords:** Beers criteria, STOPP/START criteria, Inappropriate medications, Jimma

## Abstract

**Background:**

Older adult patients are prone to potentially inappropriate medication use (PIMU); its use has been associated with multiple adverse consequences. As a result, it is crucial to determine the magnitude and factors associated with PIMU. The present study was mainly aimed to determine and assess the magnitude and predictors of potentially inappropriate medication use in older adult patients on follow-up at the chronic care clinic of Jimma medical center.

**Methods:**

A retrospective cross-sectional study was conducted involving 219 patients aged 65 years and above on treatment follow-up. Data was collected using a checklist. The 2019 updated American Geriatric Society (AGS) Beers Criteria® and Screening Tool of Older People’s Potentially Inappropriate Prescriptions criteria and Screening Tool to Alert Doctors to Right Treatment (STOPP/START) criteria (version 2) were employed to assess PIMU. SPSS IBM (v22) was used for data entry and analysis. Categorical variables were described using frequency and percentage, whereas continuous variables were described using mean with standard deviation (SD) or median with interquartile range (IQR). Logistic regression was conducted to identify predictors of PIMU.

**Results:**

The average number of medications prescribed per patient was 4.0 (IQR = 2.0). At least one PIMU was identified in 182 (83.1%) and 99 (45.2%) patients, based on Beers and STOPP criteria, respectively. Additionally, potential prescription omission (PPO) was observed in 24 (10.9%) patients. The risk of Beers PIMU was increased with age [AOR = 1.21, *p* <  **0.001**], hypertension [AOR = 4.17, *p* <  **0.001**], and polypharmacy [AOR = 14.10, *p* <  **0.001**], while a decrease in the risk was noted in patients with a diagnosis of ischemic stroke [AOR = 0.133, *p* = **0.01**] and asthma [AOR = 0.03, *p* <  **0.001**]. Using STOPP criteria, hypertension [AOR = 2.10, *p* = **0.04**], diabetes mellitus [AOR = 2.26, *p* = **0.04**], ischemic heart disease [AOR = 2.84, *p* = **0.04**], peripheral neuropathy [AOR = 10.61, *p* <  **0.001**], and polypharmacy [AOR = 6.10, *p* <  **0.001**] significantly increased the risk of PIMU.

**Conclusions:**

Regardless of the screening tool used to assess, the present study revealed PIMU in the large proportion of the participants. Multiple medication use and certain disease condition had increased the probability of PIMU. Hence, it is imperative to use screening tools for reviewing medications prescribed in older adult patients to ensure safety of medication therapy.

**Supplementary Information:**

The online version contains supplementary material available at 10.1186/s12877-021-02463-9.

## Introduction

The global proportion of the older adult population (age > 65 years) is projected to double from 703 million in 2019 to 1.5 billion in 2050 [1]. In Ethiopia, the proportion of the older adult population is increasing over time [2]; in 2019 populations aged 65 years and above were 3.52% of the country’s total age groups [3]. These age groups are usually fragile and more susceptible to drug-related problems as a result of multi-morbidity, polypharmacy, and the physiological changes that affect the kinetics and dynamics of drugs [4–6]. As a result, older adult patients are prone to PIMU, which is defined as using a drug in which the risk of an adverse event outweighs its clinical benefit [7]. Thus, medication selection in older adult patients should be made with carefulness [8].

There are multiple screening tools to assist the healthcare providers in selecting medication therapy and reduce the exposure of the older adult to PIMU. Among them, the AGS Beers Criteria® [9] and STOPP/ START are the two most widely used criteria [10]. Despite this, there is growing evidence suggesting therapeutic decisions in older adult patients are frequently suboptimal or potentially inappropriate [11].

Numerous studies have been conducted to determine the magnitude and factors associated with PIMU using various screening tools. Accordingly, the reported magnitude varies across the studies due to reasons like the type of screening tool used and others. Using Beers criteria, for instance, in a study from six European hospitals, at least one PIMU was identified in older adult patients ranging from 22.7 to 43.3% [12]**.** While, studies from United States [13] and Brazil [8] reported PIMU in 24 and 26.9% of the older adult patients, respectively. In the Middle East, several studies have reported a high prevalence of PIMU; 57.5% from Saudi Arabia [14], 62.6 and 76.0% from Qatar [15, 16], 59.6% from Lebanon [17], and 53.1% from Kuwait [18]. In Africa, one study from Nigeria [19] reported a 31% PIMU among older adult patients, while studies from Ethiopia revealed a nearly similar magnitude of PIMU; 27.72% from Gondar [20], 23% from Dessie [21], and 28.6% from Tigray [22]. Using STOPP/START, a study from Kuwait [18] reported at least one PIMU in 55.7%, while in a study from Gondar [23] PIMU was identified in 61.5% of older adult patients. Polypharmacy (taking more than or equal to 5 medications) [12, 24], Sex [24, 25]**,** and age [25, 26] were among the independent predictors of PIMU reported in studies**.**

PIMU poses a multitude of adverse consequences, such as adverse drug events [27–31]**,** an increase in healthcare expenditures [32–39], unplanned re-admission [40, 41], and an increase in mortality [42–46]. As a result, knowing the magnitude and factors that increase the risk of PIMU is important. In Ethiopia, there are limited studies. Therefore, the present study was conducted with a primary aim to determine and assess the magnitude and predictors of potentially inappropriate medication use in older adult patients on follow-up at the chronic care clinic of Jimma Medical Center (JMC).

## Methods

### The study aim, design, and setting

The primary aim of this study was to determine and assess the magnitude and predictors of potentially inappropriate medication use in older adult patients on follow-up at the chronic care clinic of Jimma Medical Center. It has also addressed the magnitude of PPOs, the internal agreement, sensitivity, and specificity of beers and STOPP criteria in detecting PIMU. A hospital-based retrospective cross-sectional study design was employed. The study was conducted in JMC chronic care clinic from November 01, 2020, to December 30, 2020. JMC is the only specialized teaching hospital in Southwest Ethiopia. It is located in Jimma town, 352 km southwest of the capital city, Addis Ababa. JMC is the only teaching and referral hospital in the South Western part of Ethiopia with a bed capacity of 620. It provides services for approximately 9000 inpatient and 80,000 outpatient clients a year with a catchment population of about 15 million people.

### Population

#### Source population

The source population was older adult patients on follow-up at the chronic care clinic of JMC.

#### Study population

Older adult patients aged 65 years and above who had a treatment follow-up at the chronic care clinic of JMC for at least six months before the current study were included in the study. Those patient charts which do not have specific necessary criteria to declare PIMU were excluded during the data collection process. Accordingly, we excluded four clinical charts due to lack of ejection fraction or serum creatinine.

### Sample size and sampling procedure

The sample size was determined by using a single population proportion formula considering the standard normal variance (Z) = 1.96; the estimated prevalence of PIMU (P) = 61.5% from the Gondar study [23], and margin of error (D) =5%; total older adult patients aged 65 and above on active follow-up in the setting (N) = 543. This had resulted in a final sample of (n) =219. The participants were selected using a systematic random sampling technique.

### Study variables

#### Outcome/dependent variables

The independent variable of this study was the presence or absence of PIMU (according to Beers criteria, STOPP/START criteria) and PPOs.

#### Independent variables

Socio-demographic variables (age, gender, marital status, residence), clinical and medication-related variables (chronic disease type and number, charlson comorbidity index score (CCI), medications regimen, number of medications per patient). CCI score was determined using the online charlson comorbidity index-MDCalc [47].

### Data collection tool and procedure

Data was collected using a checklist developed by extracting relevant variables from related literature. Two professionals (bachelor’s degree graduates in patient-oriented pharmacy) were employed as data collectors. The data collectors reviewed medical charts of older adult patients, individuals aged 65 years and above as per this study [1], to extract relevant socio-demographic and clinical information, and to establish the list of all most recent medication regimen the patient received during the last visit to the chronic care clinic.

### PIM assessment

After the completion of data collection, three clinical pharmacists (masters of clinical pharmacy graduates) assessed PIMU using the 2019 updated AGS Beers Criteria® [9] and STOPP/START criterion (Version 2) [10]. Both criteria were used in the previous studies from Ethiopia [20, 48, 49]. The AGS Beers Criteria® contains an explicit list of PIMs that are typically best avoided by older adults in most circumstances or under specific situations, such as in certain diseases or conditions. The criteria are comprised of five categories: medications that are potentially inappropriate in older adults, those that should typically be avoided in older adults with certain conditions, drugs to use with caution, drug-drug interactions, and drug dose adjustment based on kidney function. On the other hand, the STOPP/START criteria version 2 was applied to identify a list of PIMs (STOPP criteria) and Potential Prescription Omissions (PPOs) (START criteria). STOPP/START is consists of 80 STOPP and 34 START criteria. START criteria contain medications that should be considered for people with certain conditions (PPOs).

### Data quality assurance

To ensure the quality of the data, a brief training was provided to the data collectors on the objective of the study, data collection tool, and collection procedure. Before the actual data collection, a pre-test was done by reviewing eleven [11] medical charts of the older adult participants to check the validity of the checklist for most of the items of the study. PIM assessors were also made more familiar with 2019 updated AGS Beers Criteria® and STOPP/START criteria (Version 2) for assessing PIMU.

### Data processing and analysis

Data entry, clearance, and analysis was carried out using SPSS version 22.0. Frequency and percentage were calculated for categorical variables. For continuous variables, the normality test was done using the Shapiro-Wilk test; data were considered normally distributed when the *p-value* of the test is not < 0.05. Then, parametric (normally distributed) data were presented using mean, whereas median was calculated for non-parametric variables. Patients’ diagnoses were grouped according to the categories listed in the international classification of diseases-11 (ICD11) [50]. A minimal threshold of five medications was used to declare polypharmacy [51]. Spearman’s rho (r_s_) correlation test was conducted to check the presence and strength of correlation between the number of PIMs identified using Beers criteria and STOPP criteria, while cohen’s kappa (κ) test was conducted to determine the reliability between the two PIM raters used in this study (Beers criteria and STOPP criteria). The sensitivity and specificity of the two PIM raters were also checked. Using a dichotomous variable to represent the presence or absence of PIM (0 = no PIM, 1 = PIM), a binary logistic regression analysis was conducted after checking the cell adequacy of each categorical variable using a chi-square test. Variables with a *p*-value < 0.25 were recruited for multivariable logistic regression analysis. Hosmer and lemeshow test was conducted and both models of logistic regression indicated a good fit (*P* > 0.05). In all statistics, a cut-off *p-value* <  0.05 was considered to declare the statistical significance of the association.

## Results

### Overview of the study

This study involved ambulatory patients (*n* = 219) aged ≥65 years old on follow-up at the chronic care clinic for at least 6-months. The average age of the study participants was 70 (IQR = 9), and nearly two-thirds (*n* = 143; 65.3%) of the participants were male (Table 1).

**Table 1:** Sociodemographic information of the participants

Sociodemographic information	
Age, year	70 (IQR = 9)
Sex	
Male	143 (65.3%)
Female	76 (34.7%)
Residence	
Urban	106 (48.4%)
Rural	113 (51.6%)

IQR-Interquartile range.

### Clinical and related information

All of the participants had at least one chronic disease. Diseases of the circulatory system were the most common class of diseases, hypertension (*n* = 127; 58%) being the predominant of all (Table 2).

**Table 2:** Clinical and related information of the study participants

Clinical and related information		
Outpatient visits in the last 06 months		
	1–2 times	34 (15.5%)
	3 times	99 (45.2%)
	4 times	37 (16.9%)
	5 times	18 (8.2%)
	6 times	31 (14.2%)
Number of chronic diseases	One	81 (37.0%)
	Two	97 (44.3%)
	≥ Three	41 (18.7%)
CCI, mean ± SD		3.6 ± 1.1
Disease of the circulatory system		
Hypertension		127 (58%)
Ischemic heart disease		30 (13.7%)
Ischemic stroke		21 (9.6%)
Heart failure		16 (7.3%)
Hypertensive heart disease		15 (6.8%)
Ischemic dilated cardiomyopathy		15 (6.8%)
Atrial fibrillation		8 (3.7%)
Others^€^		8 (3.7%)
Chronic rheumatic valvular heart disease		6 (2.7%)
Certain infectious and parasitic diseases^¶^		5 (2.5%)
Endocrine, nutritional and metabolic diseases		
Diabetes mellitus		69 (31.5%)
Goiter		2 (0.9%)
Thyrotoxicosis		1 (0.5%)
Diseases of the nervous system		
Peripheral neuropathy		25 (11.4%)
Epilepsy		13 (5.9%)
Hemiparesis		6 (2.7%)
Others^¥^		4 (2%)
Disease of the respiratory system		
Asthma		8 (3.7%)
Chronic obstructive pulmonary disease		7 (3.2%)
Interstitial lung disease		1 (0.5%)
Disease of the digestive system		
Dyspepsia		7 (3.2%)
Chronic liver disease		1 (0.5%)
Disease of the genitourinary system		
Benign prostatic hyperplasia		3 (1.4%)
Chronic kidney disease		2 (0.9%)
Disease of the blood and blood-forming organs		
Iron deficiency anemia		1 (0.5%)
Disease of the eye and adnexa		
Glaucoma		1 (0.5%)

^¶^ Human immunodeficiency virus disease, viral hepatitis, Neurosyphilis, Pneumonia, Pulmonary tuberculosis. ^¥^Hemiplegia, Neurofibromatosis, Reye syndrome, Parkinson’s disease. ^€^Deep vein thrombosis, Degenerative valvular disease, Hemorrhagic stroke, Transient ischemic attack. CCI-Charlson comorbidity index

### Medication-related information and PIMU

The total number of prescribed medications was 902; on average each patient was prescribed 4.0 (IQ = 2.0) medications. Overall, 93.0 (42.5%) patients were on polypharmacy. PIMU was identified in 182 (83.1%) and 99 (45.2%) patients, according to beers and STOPP criteria, respectively. Furthermore, 24 (10.9%) patients had at least one PPO (Table 3).

**Table 3:** Medication-related information and the magnitude of PIMU identified in the study

Medication-related information	
Medication prescription per patient, median (IQR)	4.0 (2.0)
Patients on Polypharmacy	93 (42.5%)
According to Beers criteria	
Total PIMs	285
Patients on PIMs	182 (83.2%)
PIMs per patient, median (IQR)	1.0 (1.0)
One PIM	100 (45.7%)
Two PIMs	61 (27.8%)
Three PIMs	21 (9.6%)
Beers recommendation on the PIM	
Avoid	120 (42.1%)
Use with caution	165 (87.9%)
According to the STOPP criteria	
Total PIMs	128
Patients on PIMs	99 (45.2%)
PIMs per patient, Mean (± SD)	0.6 (±0.76)
One PIM	77 (35.2%)
Two PIMs	15 (6.8%)
Three PIMs	7 (3.2%)
PPO according to the START criteria	
Total PPOs	25
Patients with PPOs	24 (10.9%)

PIMs-Potentially inappropriate medications, PPO- Potential Prescription Omissions, STOPP-Screening Tool of Older People’s Potentially Inappropriate Prescriptions, START- Screening Tool to Alert Doctors to Right Treatment.

According to Beers criteria, aspirin (*n* = 71; 24.9%) was the most commonly prescribed PIM which needs cautious use in those aged 70 years and above followed by hydrochlorothiazide (*n* = 50; 17.5%) again with cautious use recommendation (Table 4).

**Table 4:** Specific Beers PIMs prescribed in the elderly patients involved in the study

PIM		Drug class	Frequency (%)	Recommendation	Quality of Evidence	Strength of recommendation
Independent of diagnosis	Aspirin	Anti-platelets	71(24.9)	Use with caution in adults ≥70 years	Use with caution in adults ≥70 years	Strong
	Hydrochlorothiazide	Thiazide diuretics	50 (17.5)	Use with caution	Moderate	Strong
	Amitriptyline	TCA anti-depressants	43 (15)	Avoid	High	Strong
	Furosemide	Loop diuretics	39 (13.7)	Use with caution	Moderate	Strong
	Glibenclamide	Sulphonyl urea	18 (6.3)	Avoid	High	Strong
	Omeprazole	Proton pump inhibitors	14 (4.9)	Avoid scheduled use for > 8 weeks	High	Strong
	Nifedipine	CCBs	9 (3.2)	Avoid	High	Strong
	Regular Insulin	Hormone	7 (2.5)	Avoid	Moderate	Strong
	Phenobarbital	Barbiturates	7 (2.5)	Avoid	High	Strong
	Spironolactone	Potassium sparing diuretics	6 (2.1)	Use with caution	Moderate	Strong
	Pantoprazole	Proton pump inhibitors	4 (1.4)	Avoid scheduled use for > 8 weeks	High	Strong
	Tramadol	Narcotic analgesics	3 (1.1)	Use with caution	Moderate	Strong
	Indomethacin	NSAIDs	2 (0.7)	Avoid	Moderate	Strong
	Carbamazepine	Anti-convulsant	2 (0.7)	Use with caution	Moderate	Strong
	Ibuprofen	NSAIDs	1(0.4)	Avoid chronic use	Moderate	Strong
Depend on diagnosis	Digoxin	Digitalis glycosides	3 (1.1)	Avoid this rate control agent as first line therapy for AF	AF: low Heart failure: low Dosage > 0.125 mg/day: moderate /day: strong	AF: strong Heart failure: strong Dosage > 0.125 mg
Drug-drug interaction	Enalapril + spironolactone	ACEIs and potassium sparing diuretics	6(2.1)	Use with caution in adults ≥70 years	Moderate	Strong
		**Total PIM**	285(100)			

CCBs-Calcium channel blockers, ACEIs ACEIs-Angiotensin converting enzyme inhibitors, NSAID-Non-steroidal anti-inflammatory drug, AF-Atrial fibrillation.

Using STOPP criteria, the most commonly prescribed PIM was amitriptyline (*n* = 38; 29.7%) followed by furosemide (*n* = 27; 21%) and glibenclamide (*n* = 18; 14%). Whereas, the most commonly omitted medication observed were ACE inhibitors (58.3%), followed by beta-blockers (29.2%) and aspirin (4.2%) (Table 5).

**Table 5:** Specific PIMs and PPOs according to STOPP/START criteria

PIM	Drug class	Frequency (%)
**Using STOPP criteria**		
Amitriptyline	TCA anti-depressants	38 (29.7)
Furosemide	Loop diuretics	27 (21)
Glibenclamide	Sulphonyl urea	18 (14)
Enalapril	ACEIs	12 (9.4)
Hydrochlorothiazide’s	Thiazide diuretics	10 (7.8)
Aspirin	Anti-platelet	8 (6.25)
Metformin	Biguanides	4 (3.1)
Clopidogrel	Anti-platelet	3 (2.3)
Digoxin	Digitalis glycosides	2(1.6)
Tramadol	Narcotic analgesics	2(1.6)
Metoprolol	Beta blocker	2(1.6)
Indomethacin	NSAID	1(0.8)
Meloxicam	NSAID	1(0.8)
**Total**		**128 (100)**
**Using START criteria (PPOs)**		
ACEIs	ACEIs	14(58.3)
Beta-blockers	Beta-blockers	7(29.2)
Aspirin	Anti-platelet	1(4.2)
Non-TCA anti-depressants	Non-TCA antidepressants	1(4.2)
Regular inhaled beta 2 agonist	Regular inhaled beta 2 agonist	1(4.2)

ACEIs-Angiotensin-converting enzyme inhibitors, TCA-Tricyclic antidepressants, NSAID-Non-steroidal anti-inflammatory drug.

### Correlations, reliability, sensitivity, and specificity of PIM raters used in this study

The two PIM raters used in this study i.e., Beers and STOPP criteria, had a minimal and inadequate agreement in rating PIMs (κ = 0.22, 95%CI: 0.15, 0.31, *p* = < **0.001**). Additionally, the number of PIMs identified using these two criteria were also fairly correlated with each other (r_s_ = 0.48, *p* = < **0.001**). Presuming STOPP criteria as a test result, it had a sensitivity of 52.20% and specificity of 89.19%, whereas taking beers criteria as a test result, the sensitivity and specificity of beers criteria was 96.0 and 27.5%, respectively.

### Factors associated with PIMU based on Beers’ criteria

On binary logistic regression, age (*p* <  **0.001**), frequency of outpatient visits in the last six months [four times (*p* = **0.03**) and six times (*p* = **0.02**)], hypertension (*p* <  **0.001**), asthma (*p* = **0.02**), epilepsy (*p* <  **0.001**), and polypharmacy (*p* <  **0.001**) were significantly associated with Beer’s PIM. A total of eight variables had a *p-*value < 0.25 and were recruited for multivariate logistic regression. Upon conducting a multivariate logistic regression, age [AOR = 1.21, 95%CI: 1.09, 1.34, *p* <  **0.001**], hypertension [AOR = 4.17, 95%CI: 1.51, 11.56, *p* <  **0.001**], ischemic stroke [AOR = 0.133, 95%CI: 0.03, 0.64, *p* = **0.01**], asthma [AOR = 0.03, 95%CI: 0.00, 0.39, *p* <  **0.001**], and polypharmacy [AOR = 14.10, 95%CI: 2.61, 76.38, *p* <  **0.001**] were independently associated with Beer’s PIM (Table 6).

**Table 6:** Logistic regressions analysis for identifying predictors of Beers PIMU

Variables		PIM users	PIM non-users	COR [95%CI]	***p***-value	AOR [95%CI]	***p***-value
Age, year		70 (IQR = 9)	67 (IQR = 5)	1.15 [1.06, 1.26]	< 0.00	1.21 [1.09, 1.34]	**< 0.001**
Sex	Male	118 (64.8%	25 (67.6%)	0.89 [0.42, 1.88]	0.75	–	
	Female	64 (35.2%)	12 (32.4%)	1			
Residence	Urban	91 (50.0%)	15 (40.5%)	1.47 [0.72, 3.01]	0.29	–	
	Rural	91 (50.0%)	22 (59.5%)	1			
Outpatient visits in the last 06 months							
	1–2 times	23 (12.6%)	11 (29.7%)	1		1	
	3 times	81 (44.5%)	18 (48.7%)	2.15 [0.89, 5.19]	0.09	2.55 [0.80, 8.14]	0.11
	4 times	33 (18.1%)	4 (10.8%)	3.95[1.11, 13.94]	0.03	3.48 [0.69, 17.52]	0.13
	5 times	16 (8.8%)	2 (5.4%)	3.83 [0.75, 19.65]	0.11	3.53 [0.48, 26.05]	0.22
	6 times	29 (15.9%)	2 (5.4%)	6.94 [1.39, 34.45]	0.02	5.07 [0.74, 34.71]	0.09
Number of chronic diseases							
	One	59 (32.4%)	22 (59.5%)	1		1	
	Two	89 (48.9%)	8 (21.6%)	0.55 [0.21, 1.43]	0.22	2.70 [0.85, 8.61]	0.09
	≥ Three	34 (18.7%)	7 (18.9%)	2.29 [0.77, 6.80]	0.14	1.46 [0.29, 7.28]	0.65
Hypertension	Yes	117 (64.3%)	10 (27.0%)	4.86 [2.21, 10.67]	< 0.00	4.17 [1.51, 11.56]	**< 0.001**
	No	65 (35.7%)	27 (73.0%)	1		1	
Diabetes mellitus	Yes	60 (33.0%)	9 (24.3%)	1.53 [0.68, 3.45]	0.31		
	No	122 (67.0%)	28 (75.7%)	1			
Ischemic heart disease	Yes	26 (14.3%)	4 (10.8%)	1.35 [0.45, 4.20]	0.58		
	No	156 (85.7%)	33 (89.2%)	1			
Ischemic stroke	Yes	15 (8.2%)	6 (16.2%)	0.46 [0.17, 1.29]	0.14	0.133 [0.03, 0.64]	**0.01**
	No	167 (91.8%)	31 (83.8%)	1		1	
Asthma	Yes	4 (2.2%)	4 (10.8%)	0.19 [0.04, 0.78]	0.02	0.03 [0.00, 0.39]	**< 0.001**
	No	178 (97.8%)	33 (89.2%)	1		1	
Heart failure	Yes	15 (8.2%)	1 (2.7%)	3.23 [0.41, 25.27]	0.26		
	No	167 (91.8%)	36 (97.3%)	1			
Hypertensive heart disease	Yes	11 (6.0%)	4 (10.8%)	0.53 [0.16, 1.77]	0.53		
	No	171 (94.0%)	33 (89.2%)	1			
Peripheral neuropathy	Yes	25 (13.7%)	0		0.99		
	No	157 (86.3%)	37 (100.0%)	1			
Epilepsy	Yes	7 (3.9%)	6 (16.2%)	0.21 [0.07, 0.66]	< 0.00	0.42 [0.09, 2.08]	0.29
	No	175 (96.1%)	31 (83.8%)	1		1	
Chronic obstructive pulmonary disease	Yes	5 (2.8%)	2 (5.4%)	0.49 [0.09, 2.65]	0.41		
	No	177 (97.2%)	35 (94.6%)	1			
Dyspepsia	Yes	6 (3.3%)	1 (2.7%)	1.23 [0.14, 10.51]	0.85		
	No	176 (96.7%)	36 (97.3%)	1			
Ischemic dilated cardiomyopathy	Yes	14 (7.7%)	1 (2.7%)	3.0 [0.38, 23.55]	0.29		
	No	168 (92.3%)	36 (97.3%)	1			
Atrial fibrillation	Yes	7 (3.8%)	1 (2.7%)	1.44 [0.17, 12.07]	0.74		
	No	175 (96.2%)	36 (97.3%)	1			
Polypharmacy	≥ 5 drugs	89 (48.9%)	4 (10.8%)	7.89 [2.69, 23.19]	< 0.00	14.10 [2.61, 76.38]	**< 0.001**
	< 5 drugs	93 (51.1%)	33 (89.2%)	1		1	

### Factors associated with PIMU based on STOPP criteria

On binary logistic regression, the variables: above two times outpatient visits in the last 6-months, number of chronic diseases, hypertension, diabetes mellitus, ischemic heart disease**,** peripheral neuropathy, and polypharmacy were significantly associated with PIM use based on STOPP criteria. Running multiple logistic regression, hypertension [AOR = 2.10 48, 95%CI: 1.04, 4.29, *p* = **0.04**], diabetes mellitus [AOR = 2.26, 95%CI: 1.037, 4.91, *p* = **0.04**], ischemic heart disease [AOR = 2.84, 95%CI: 1.05, 7.67, *p* = 0.04], peripheral neuropathy [AOR = 10.61, 95%CI: 3.08, 36.54, *p* <  **0.00**1], and polypharmacy [AOR = 6.10, 95%CI: 3.08, 14.59, *p* <  **0.00**1] significantly increased the risk of using PIM (Table 7).

**Table 7:** Logistic regressions analysis for identifying predictors of PIMU based on STOPP criteria

Variables		PIM users	PIM non-users	COR [95%CI]	***p***-value	AOR [95%CI]	***p***-value
Age, years		70 (IQ = 9)	70 (IQ = 10)	1.01 [0.97, 1.06]	0.64	–	
Sex	Male	64 (64.6%)	79 (65.8%)	0.95 [0.54, 1.66]	0.85	–	
	Female	35 (35.4%)	41 (34.2%)	1			
Residence	Urban	49 (49.5%)	57 (47.5%)	1.08 [0.64, 1.85]	0.77	–	
	Rural	50 (50.5%)	63 (52.5%)	1			
Outpatient visits in the last 06 months							
	1–2 times	9 (9.1%)	25 (20.8%)	1		1	
	3 times	39 (39.4%)	60 (50.0%)	1.81 [0.76, 4.28]	0.18	0.77 [0.27, 2.15]	0.61
	4 times	24 (24.2%)	13 (10.8%)	5.13[0.76, 14.19]	< 0.00	1.63 [0.48, 5.57]	0.44
	5 times	11 (11.1%)	7 (5.8%)	4.37 [1.85, 14.73]	0.02	2.62 [0.64, 10.73]	0.18
	6 times	16 (16.2%)	15 (12.5%)	2.96 [1.29, 8.36]	0.04	0.85 [0.24,3.00]	0.79
Number of Chronic diseases							
	One	23 (23.2%)	58 (48.3%)	1		1	
	Two	52 (52.5%)	45 (37.5%)	2.91 [1.56, 5.45]	< 0.00	0.79 [0.34, 1.80]	0.57
	≥ Three	24 (24.2%)	17 (14.2%)	3.56 [1.62, 7.82]	< 0.00	0.48 [0.16, 1.48]	0.20
Hypertension	Yes	68 (68.7%)	59 (49.2%	2.27 [1.30, 3.95]	< 0.00	2.10 [1.04, 4.29]	**0.04**
	No	31 (31.3%)	61 (50.8%)	1		1	
Diabetes mellitus	Yes	47(47.5%)	22 (18.3%	4.03 [2.19, 7.39]	< 0.00	2.26 [1.037, 4.91]	**0.04**
	No	52 (52.5%)	98 (81.7%)	1		1	
Ischemic heart disease	Yes	18 (18.2%)	12 (10.0%)	2.0 [0.91, 4.39]	0.08	2.84 [1.05, 7.67]	**0.04**
	No	81 (81.8%)	108 (90.0%)	1		1	
Ischemic stroke	Yes	7 (7.1%)	14 (11.7%)	0.57 [0.22, 1.49]	0.25		
	No	92 (92.9%)	106 (88.3%)	1			
Heart failure	Yes	6 (6.1%)	10 (8.3%)	0.71 [0.25, 2.03]	0.52		
	No	93 (93.9%)	110 (91.7%)	1			
Hypertensive heart disease	Yes	7 (7.1%)	8 (6.7%)	1.07 [0.37, 3.05]	0.91		
	No	92 (92.9%)	112 (93.3%)	1			
Peripheral neuropathy	Yes	20 (20.2%)	5 (4.2%)	5.82 [2.09, 16.16]	< 0.00	10.61 [3.08, 36.54]	**< 0.001**
	No	79 (79.8%)	115 (95.8%)	1			
Epilepsy	Yes	0	13 (10.8%)	0.0	0.99		
	No	99(100.0%)	107 (89.2%)	1			
Ischemic dilated cardiomyopathy	Yes	8 (8.1%)	7 (5.8%)	1.42 [0.49, 4.06]	0.51		
	No	91 (91.9%)	113 (94.2%)	1			
Polypharmacy	< 5 drugs	67 (67.7%)	26 (21.7%)	1		1	
	≥ 5 drugs	32 (32.3%)	94 (78.3%)	7.57 [4.13, 13.86]	< 0.00	6.10 [3.08, 14.59]	**< 0.001**

## Discussion

This was a retrospective cross-sectional study conducted involving 219 older adult patients on follow-up at the chronic care clinic of a specialized teaching medical center in Ethiopia. The main objective of this study was to determine the magnitude and factors associated with PIMU based on Beers and STOPP criteria. Accordingly, 83.2 and 45.2% of the patients had at least one PIM based on Beers and STOPP criteria, respectively. Additionally, 24 (10.9%) patients had at least one PPO.

In the present study, the magnitude of PIMU based on Beers criteria was higher than in some previous studies. The magnitude of PIMU was 50.0% in a study from USA [52], 26.9% from Brazil [8], and 30.5% from Irish [24]. In India, studies had reported PIMU prevalence of 23.5% [53], 24.6% [26], and 61.9% [25], while in studies from middle east, PIMU was reported in 53.1% from Kuwait [18]; 61.0% from Saudi Arabia [27]; 45.2% from Lebanon [54]; 62.6% [15] and 76.0% [16] from Qatar. In Africa, studies are limited. One study from Nigeria [19] reported a 31% of PIMU, while in Ethiopia, studies from Tigray [22]; Gondar [20], and Dessie [21] reported PIMU in 28.6 27.7, and 23% of the older adult patients, respectively. The discrepancy in the magnitude of PIMU could be due to many factors. For instance, Beers criteria are the commonly used guidelines to manage and improve the care of individual aged 65 years and older adult in healthcare settings [9]. Contrary to this, our study setting lacks the privilege of flagging potentially inappropriate medication lists for extra caution which will make prescribers comfortably rely on the same medication for years without the concern of safety. Additionally, adopting a different version of Beers criteria in the previous studies (AGS Beers Criteria 2012 and 2015) as compared to the present study (AGS Beers Criteria 2019) might also explain the difference in the magnitude of PIMU. Furthermore, the difference in the data collection method (chart review versus prospective) employed across those studies might have also contributed to the variation in the magnitude of PIMU.

Based on STOPP criteria, at least one PIMU was identified in 45.2% of the older adult patients in our study. This indicates nearly half of our participants were taking medication that could be harmful to their health. Using the same criteria, studies from Gondar [23] and Kuwait [18] reported at least one PIMU in 61.5 and 55.7% of older adult outpatients, respectively. These magnitudes are higher than our study finding. The Gondar study was a prospective study which is a better design to track all medication used by the patient, and the Kuwait study was also a prospective study, and the investigators employed both medical electronic and non-electronic records to exhaustively access the patients` prescribed medications and other information. In our case, there is only a non-electronic record (patient medical chart) to access prescribed medications and other information which might have some incomplete medication list. Besides, the limited availability of some medications in Ethiopia could have contributed to the less magnitude of PIM identified in our study.

In the present study, as the age of the patient increased, the risk of Beers PIMU was also observed to increase [*p* <  **0.001**]. Based on either Beers or STOPP criteria, hypertension and taking polypharmacy were significantly increased the probability of PIMU. Taking polypharmacy had increased the risk of PIMU by more than fourteen [*p* <  **0.001**] and six times [*p* <  **0.001**] based on Beers and STOPP criteria, respectively. Being hypertensive increased the likelihood of PIMU by more than four times [*p* <  **0.01**] and two times [*p* <  **0.04**] based on Beers and STOPP criteria, respectively.

Similarly in the previous studies, taking polypharmacy [14, 55, 56], advanced age [25, 56], and hypertension [57] were reported as significant predicting factors for PIMU. As the age advance, metabolic changes and decreased drug clearance, and increased drug-drug interactions are expected [58]. On the other hand, simultaneous use of multiple medications probably increases the risk of drug-drug, drug-disease interactions as well as diverting clinician’s attention to provide quality care, which in turn increases the likelihood of prescribing PIMs. Contrary to our expectations, in the current study, patients with ischemic stroke [*p* = **0.01**] and asthma [*p* <  **0.001**] were associated with lower Beer’s PIMU. In our study, the proportion of the patients with these disease conditions were small which could be a possible justification.

According to STOPP criteria, increased likelihood of PIMU was also observed in patients with ischemic heart disease [*p* <  **0.04**], diabetes mellitus [*p* <  **0.04**], and peripheral neuropathy [*p* <  **0.001**]. Other studies had also reported similar predictors [14–16]. Surprisingly, age was not a significant predictor of PIMU based on STOPP criteria. As chronic morbidities are expected to increases with age, so does the risk of multiple comorbidities and multiple medication use.

### Strength and limitations of the study

To our knowledge the present study is the first to identify PIMU among older adult patients concurrently using two screening tools in the healthcare setting in Ethiopia. This will enable healthcare practitioners in Ethiopia to have an insight into the sensitivity and specificity of the two most commonly used PIM assessing tools in the Ethiopian context. In the present study, PIMU identified based on either tool were adjusted for the accessible important confounders to point out the effect size of the explanatory variables. Furthermore, both PIMU assessing tools were the latest version during the time of the present study. Despite the aforementioned strengths, which could also describe the novelty, this study also has limitations that include: the small sample size and consideration of only a single institution could affect the generalizability and power of the study in identifying factors associated with PIMU. Additionally, the retrospective nature of the study has hindered confirming the actual consumption of PIMs by patients and their actual clinical consequences. Lastly, the possibility of non-documentation of medications in the patient chart such as over-the-counter medications might have underestimated the magnitude of PIMU.

## Conclusion

In the present study, PIMU was identified in a large proportion of the participants. Multiple medication use and certain comorbidities had increased the probability of PIMU. Hence, the authors recommend the use of screening tools for reviewing medications prescribed for older adult patients to reduce the adverse consequences related to PIMU. Furthermore, a multicenter, prospective, and powered study is recommended to gain more insight into the medication use among older adult patients and its impacts in health care settings in Ethiopia.

### Acronyms

ACEIs-Angiotensin-converting enzyme inhibitors, AF-Atrial fibrillation, AGS- American Geriatric Society, CCBs-Calcium channel blockers, CCI-Charlson comorbidity index, κ-Cohen’s kappa, HCT Hydrochlorothiazide, ICD11-International Classification of Diseases-11, JMC- Jimma Medical Center, IQR- Interquartile range, NSAID-Non-steroidal anti-inflammatory drug, PIMU-Potentially inappropriate medication use, PPO-Potential Prescription Omissions, r_s-_Spearman’s rho, STOPP-Screening Tool of Older People’s Potentially Inappropriate Prescriptions, START- Screening Tool to Alert Doctors to Right Treatment., SD-standard deviation, TCA-Tricyclic antidepressants.

## Supplementary Information

**Additional file 1: S1 file.** PIM data dataset.xlsx.

**Additional file 2: S2 file.** PIM data dataset.xlsx.
